# Attitudes of European physicians towards the use of long-acting injectable antipsychotics

**DOI:** 10.1186/s12888-020-02530-2

**Published:** 2020-03-14

**Authors:** Maxine X. Patel, Nawal Bent-Ennakhil, Christophe Sapin, Sylvie di Nicola, Jean-Yves Loze, Anna-Greta Nylander, Stephan Heres

**Affiliations:** 1 Department of Psychosis Studies, Institute of Psychiatry, Psychology and Neuroscience, King’s College London, London, UK; 2 Lundbeck SAS, Issy-les-Moulineaux, France; 3 Inferential, 35 Rue Godot de Mauroy, 75009 Paris, France; 4 Otsuka Pharmaceutical Europe Ltd, Wexham, UK; 5 H. Lundbeck A/S, Ottiliavej 9, 2500 Valby, Denmark; 6 Department of Psychiatry and Psychotherapy, Munich Technical University, Munich, Germany

**Keywords:** Antipsychotic agents, Physicians, Attitude, Cross-sectional studies, Long-acting injectables

## Abstract

**Background:**

Prescription rates for long-acting injectable (LAI) antipsychotic formulations remain relatively low in Europe despite improved adherence over alternative oral antipsychotic treatments. This apparent under-prescription of LAI antipsychotics may have multiple contributing factors, including negative mental health practitioner attitudes towards the use of LAIs.

**Methods:**

The Antipsychotic Long acTing injection in schizOphrenia (ALTO) non-interventional study (NIS), conducted across several European countries, utilised a questionnaire that was specifically designed to address physicians’ attitudes and beliefs towards the treatment of schizophrenia with LAI antipsychotics. Exploratory principal component analysis (PCA) of feedback from the questionnaire aimed to identify and characterize the factors that best explained the physicians’ attitudes towards prescription of LAIs.

**Results:**

Overall, 136/234 solicited physicians returned fully completed questionnaires. Physicians’ mean age was 48.5 years, with mean psychiatric experience of 20.0 years; 69.9% were male, 84.6% held a consultant position, and 91.9% had a clinical specialty in general adult care. Most physicians considered themselves to have a high level of clinical experience with LAI antipsychotics (77.2%), with an increased rate of LAI antipsychotics prescription over the last 5 years (59.6%). Although the majority of physicians (69.9%) declared feeling no difference in stress levels when offering LAI compared to oral antipsychotics, feelings of ‘no/more stress’ versus ‘less stress’ was found to influence prescription patterns. PCA identified six factors which collectively explained 66.1% of the variance in physician feedback. Multivariate analysis identified a positive correlation between physicians willing to accept usage of LAI antipsychotics and the positive attitude of colleagues (co-efficient 3.67; *p* = 0.016).

**Conclusions:**

The physician questionnaire in the ALTO study is the first to evaluate the attitudes around LAI antipsychotics across several European countries, on a larger scale. Findings from this study offer an important insight into how physician attitudes can influence the acceptance and usage of LAI antipsychotics to treat patients with schizophrenia.

## Background

Antipsychotic medications play a vital role in controlling symptoms of psychosis and minimizing the likelihood of relapse, [1] and yet patient non-adherence to oral first- and second-generation antipsychotics (FGAs and SGAs, respectively) is one of the main barriers to relapse prevention [2–4]. Although adherence rates have been shown to be moderately higher in patients receiving SGAs versus FGAs, interventions for adherence improvements are warranted for both classes of antipsychotic [5, 6].

Long-acting injectable (LAI) antipsychotics were developed in the 1960s to improve adherence as they provide slow, sustained release of active drug, which reduces the frequency of administration to once or twice monthly [7]. SGA-LAI antipsychotics have been available in Europe since 2002 [8] and more recently formulations covering longer time periods are also available [2, 9, 10]. Crucially, LAI antipsychotic treatments enable healthcare providers to identify and follow-up promptly in cases of non-adherence [7].

Evidence from observational studies suggests LAI formulation antipsychotics improve medication adherence in comparison to oral treatment, [11–17] but evidence from randomised controlled trials (RCTs) suggests no difference [18–21]. The intensity of follow-up in RCTs may obscure the advantages of LAI over orals regarding compliance, but also these conflicting findings may be a consequence of the methodological rigor (including strict selection criteria) of RCTs over observational studies (i.e. elimination of bias through patient randomization), which may minimize differences between LAI and oral formulations that would otherwise manifest in clinical practice [7, 22]. Conversely, observational studies may be more generalizable to clinical practice as they can include patients who are less likely to consent to or be eligible to participate in an RCT, but they do not control for confounding factors and remain vulnerable to an element of selection bias [22].

Prescription rates of LAI antipsychotics for patients with schizophrenia vary between European countries but are generally lower than 30%, [23, 24] leading to concerns from some experts that LAI antipsychotics are under-prescribed [25]. Additionally, the registration of oral SGA antipsychotics has led to a decrease in prescribing of FGA-LAIs [26]. Reasons for the limited usage of LAI antipsychotics appear to include negative physician and patient attitudes towards this type of treatment [2, 27, 28]; as well as beliefs that LAI antipsychotics are more expensive and cause more side effects, with greater severity, than their oral counterparts [2]. Many practitioners also perceive LAIs as unlikely to be accepted by patients [2, 25, 27, 29].

Research characterizing attitudes of health professionals towards LAI antipsychotics is limited [7, 30]. Links between attitude and treatment practice are under-researched, and studies have been hampered by small sample size, flawed methodology, single country assessment and/or a poorly generalisable sample of practitioners [2]. As attitudes of health professionals are recognised to play an important role in whether patients take antipsychotics, [31] further research in this area is clinically important.

One of the objectives of the ALTO (Antipsychotic Long acTing injection in schizOphrenia) study was to evaluate current attitudes and beliefs of European physicians towards the treatment of patients diagnosed with schizophrenia with LAI antipsychotics. A physician questionnaire, with exploratory principal component analysis (PCA) of the responses, was used to identify and characterize the factors that best explained physicians’ attitudes towards prescription of FGA- and SGA-LAI antipsychotics. The findings from this study will help to facilitate more evidence-based consideration of the role for LAI antipsychotics in the treatment of patients with schizophrenia.

## Methods

### Study design

The ALTO study, a multinational, multicenter study spanning six European countries (Austria, France, Germany, Spain, Sweden, and the UK) was the first large-scale non-interventional study (NIS) in Europe to focus on LAI antipsychotics treatment of patients with schizophrenia, and has been described in detail elsewhere [32].

Retrospective, cross-sectional data was collected from patients already receiving treatment with LAI antipsychotics (prevalent users) or initiating a treatment with a LAI antipsychotic that was not prescribed during the previous 12 months (incident users). Data for the cross-sectional part of the study was collected between 5th July 2013 and 30th June 2014 (first and last patient visits, respectively). Incident users of LAI antipsychotics were followed up in a prospective, longitudinal study component over an 18-month period.

The physician population invited to answer the questionnaire included all physicians initiated to the ALTO study.

### Physician questionnaire

A physician questionnaire, investigating attitudes and beliefs concerning LAI antipsychotics, was designed for use at baseline of the ALTO study. Physicians who had completed the questionnaire fully, and who answered “Yes” to the question: “Willingness to voluntarily complete the questionnaire” were included in the analyses. Cultural differences and allowances for differences between FGA- and SGA-LAI antipsychotics were considered. Physicians participated on a voluntary basis and consent was deemed implicit upon completion of the questionnaire.

The questionnaire consisted of 11 sections, each including multiple statements or items, as well as a free answer section inviting additional comments. Sections included: sociodemographic characteristics (9 items including age, gender, country of practice, years of psychiatry experience, position, clinical specialty), clinical experience (6 items regarding experience with and prescribing habits of LAI antipsychotics), the formulation process pathway (explained in more detail below, 2 sections of 4 items each), 3 sections on LAI acceptance (1 item each regarding the level of acceptance of LAI as a treatment option for schizophrenia for each of physicians’ patients, their family members, and for themselves respectively), efficacy (3 items on how aspects of efficacy affect the decision to initiate or not initiate LAI treatment), to what extent side effects (15 items) affect the decision to initiate or not initiate LAI treatment, and to what extent general aspects (32 items) affect the decision to initiate or not initiate LAI treatment. Physicians were also asked to what extent they agreed with 7 statements about LAI medications.

Physicians scored the sections on side effects, efficacy, and general aspects on a 1–7 scale (1 = “markedly pro LAI”, 2 = “sometimes pro LAI”, 3 = “seldom pro LAI”, 4 = “neither against nor pro LAI”, 5 = “seldom against LAI”, 6 = “sometimes against LAI”, 7 = “markedly against LAI”) to establish the degree to which these factors influenced initiation of LAI antipsychotics treatment.

Physicians’ consideration of oral or LAI antipsychotic formulations when prescribing a new antipsychotic was investigated by asking them to declare the proportion of their patients with schizophrenia for which they: “consciously think about an [oral formulation/LAI]”, “discuss the option of an [oral formulation/LAI] with the patient”, “try really hard to gain consent for an [oral formulation/LAI]”, and “actually prescribe an [oral formulation/LAI]”. These four steps (think, discuss, attempt consent, prescribe) were collectively termed the formulation process pathway.

Physicians were also asked “In general, is there a difference in how much stress you feel when offering LAI to a patient compared to oral treatment?” Responses were provided on a 5-point scale (“much more stressed,” “a little more stressed,” “no difference,” “a little less stressed,” and “much less stressed”).

### Descriptive statistics and analysis of variance

All data analysis for this paper was generated using SAS software, Version 9.2 of the SAS System for Copyright© 2009 (SAS Institute Inc., Cary, NC, USA).

Physician questionnaire responses on the 5-point stress-level scale were aggregated into two categories: physicians who felt “less stress” (those answering “much less stressed” were grouped with those answering “a little less stressed”), and physicians who felt “no or more stress” (those answering “a little more stressed” were grouped with those answering “no difference in stress”). A Student’s *t*-test was used to compare the mean difference in the proportion of patients (oral - LAI) reported by physicians according to the two stress categories (“less stressed” vs “no or more stressed”) at each step of the formulation process pathway.

To identify variables associated with or potentially influencing the prescription of antipsychotics, a multiple regression analysis (repeated measure ANOVA model) was performed, with the physician-reported percentages of patients with schizophrenia for whom an antipsychotic would be prescribed as the outcome variable. The ANOVA model included proportion of patients as a response variable, formulation and stress as factors, the interaction between formulation and stress, and the formulation process pathway as a repeated factor as each physician provided answer for all four stages of the pathway and therefore the stages were related.

Physicians rated the influence of potential side effects according to the question: “To what extent do the following side effects affect your decision to initiate (or not initiate) LAI treatment”, with results converted to the 1–7 scale. Summary statistics, including the mean category score and 95% confidence intervals, were computed for all potential side effects.

### Principal component analysis and regression analysis

PCA was conducted on 34 items from the efficacy and general aspects sections of the questionnaire, with the aim of identifying and characterizing the factors that best explained physicians’ attitudes towards prescription of LAIs. A varimax orthogonal rotation was used and the Kaiser-Meyer-Olkin value was computed to measure sample adequacy (values > 0.8 confirmed adequacy). The number of factors was informed by considering a minimum eigenvalue of 1, a minimum factor loading of 0.4, and the proportion of variance explained (cumulative variance > 50%). Factor internal consistency was assessed with Cronbach’s alpha. The 7-point scale of measurement was aggregated to “Disagree” = 5–7 (“seldom against LAI”, “sometimes against LAI”, and “markedly against LAI”), “Neither agree nor disagree”= 4 (“neither against nor pro LAI”), and “Agree” = 1–3 (“markedly pro LAI”, “sometimes pro LAI”, and “seldom pro LAI”), to describe the items pertaining to the identified factors.

Univariate regression analysis was used to determine what variables affected the proportion of patients that physicians declared willing to accept LAI antipsychotics treatment. Factors resulting from the PCA, and other variables relating to physician characteristics (age, gender, specialty, clinical setting, number of years’ experience, and country) were considered. Variables found to be associated in the univariate analysis (*p* < 0.05) were included in the final multivariate linear regression model.

## Results

### Physician disposition and sociodemographic characteristics

Overall, 177/234 solicited physicians returned their questionnaire (75.6%); 136 of the questionnaires (76.8%) were complete and were included in the analysis set. The majority of the physicians were male (69.9%), were in a consultant position (84.6%), and had a clinical speciality in general adult care (91.9%). The highest proportion of physicians were from the UK (31.6%); Table 1. Mean physician age was 48.5 years, with mean years of psychiatry experience after medical qualification of 20.0 years. Most physicians practiced within their National Health Service (NHS) or a university clinic setting (59.6%).

**Table 1:** Physician Sociodemographics and Clinical Experience

Characteristic	Physicians, ***n*** (%) (***N*** = 136^**a**^)
**Clinician status**	
Resident/Trainee	19 (14.0)
Consultant	115 (84.6)
Other/Missing	2 (1.5)
**Country**	
UK	43 (31.6)
Germany	35 (25.7)
Austria	2 (1.5)
France	23 (16.9)
Spain	30 (22.1)
Sweden	3 (2.2)
**Setting**	
NHS only	37 (27.2)
Private practice only	24 (17.6)
Both NHS and private practice	6 (4.4)
University clinic	44 (32.4)
Other clinic	25 (18.4)
**In your opinion, does your country have a history of large scale use of first generation LAI prescriptions (> 20% of all antipsychotic prescriptions)?**	
Yes, and this is still the case	30 (22.1)
Yes, but this number has dropped	80 (58.8)
Not historically, but this is now the case	8 (5.9)
No, this was never the case	18 (13.2)
**Has your rate of prescribing LAI antipsychotics for schizophrenia changed over the last 5 years?**	
Marked decrease	2 (1.5)
Slight decrease	16 (11.8)
Unchanged	37 (27.2)
Slight increase	55 (40.4)
Marked increase	26 (19.1)
**How would you rate your level of clinical experience with the use of antipsychotics LAIs for schizophrenia?**	
No experience	1 (0.7)
Minimal experience	2 (1.5)
Somewhat experienced	28 (20.6)
Very experienced	105 (77.2)

^a^177/234 solicited physicians returned a questionnaire. 136 were completed in full, 35 were excluded because they were incomplete, 5 were excluded because the physician answered no to the section “Willingness to voluntarily complete the questionnaire”, and 1 was excluded because the answer to the section “Willingness to voluntarily complete the questionnaire” was missing. LAI, long-acting injectable; NHS, National Health Service

Sociodemographic and clinical experience data (Table 1) revealed that most physicians considered their country to have a history of large scale FGA-LAI use, i.e. accounting for > 20% of all antipsychotics prescriptions (80.9%), and the majority of these felt the number of FGA-LAI prescriptions had dropped (58.8%). Most physicians considered themselves to have a high level of clinical experience with LAI antipsychotics (77.2%), and that their rate of LAI antipsychotics prescriptions over the last 5 years had increased (59.6%).

### Formulation process pathway

**Fig. 1:**
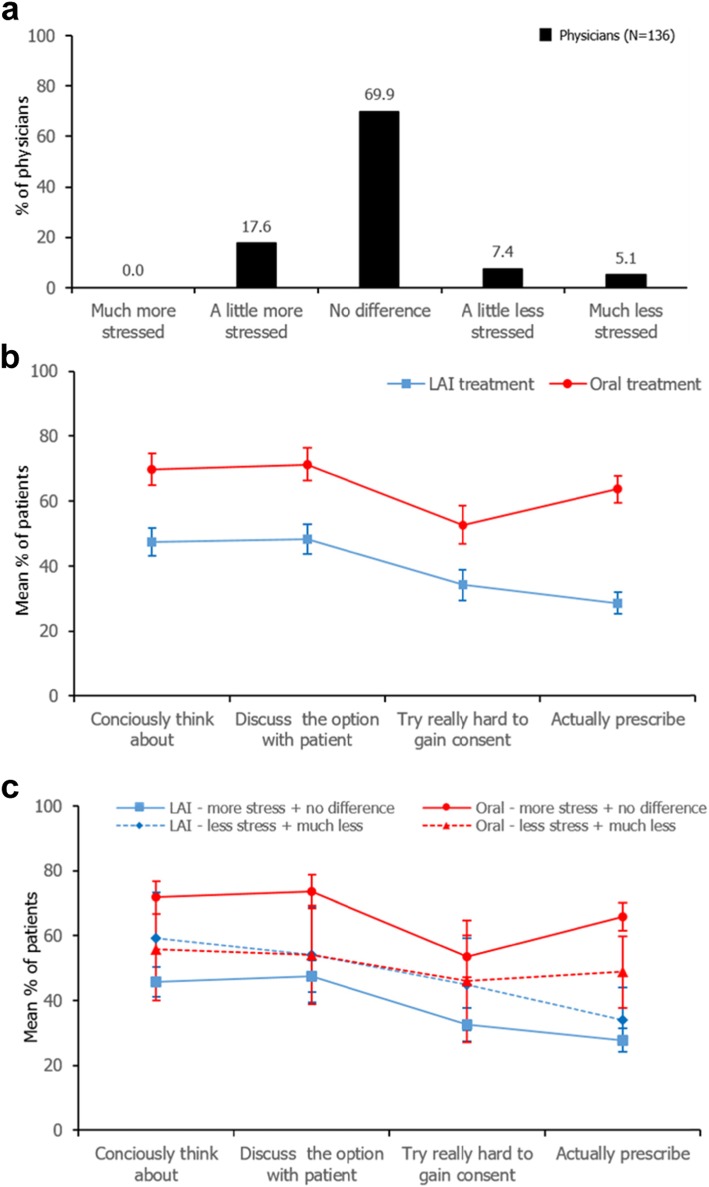
Stress Levels and Formulation Process Pathway for Physicians Offering Long-Acting Injectable Antipsychotics. **a** Physicians’ stress levels when offering LAI over oral treatment - proportions of physicians who rated their level of stress as felt when offering LAI over oral antipsychotic formulations to patients with schizophrenia are shown. **b** Formulation process pathway: prescription of LAI vs oral treatment - proportions of patients that physicians declared for each stage of the formulation process pathway when prescribing a new antipsychotic and considering oral versus LAI antipsychotic formulations. Mean ± 95% CI is shown. **c** Formulation process pathway by stress level - the influence of stress associated with offering LAI compared to oral treatments on proportions of patients that physicians declared for each stage of the formulation process pathway. Mean ± 95% CI is shown. CI, confidence interval; LAI, long-acting injectable

The majority of physicians (69.9%) declared feeling no difference in stress levels when offering LAI compared to oral antipsychotic formulations. Of the remaining physicians, 17.6% declared feeling a little more stressed, and 12.5% less stressed (Fig. 1a). When the formulation process pathway was examined, physicians viewed oral versus LAI antipsychotic formulations (mean ± standard deviation) at each of the four steps as: think about (oral 69.7 ± 28.2%, LAI 47.4 ± 25.3%), discuss (oral 71.3 ± 29.1%, LAI 48.2 ± 27.1%), attempt consent (oral 52.7 ± 34.5%, LAI 34.1 ± 27.8%), and prescribe (oral 63.7 ± 23.6%, LAI 28.6 ± 19.8%). The gap between LAI and oral treatment was greatest at the prescribe stage, indicating greater consideration over treatment options at this stage of the formulation process pathway (Fig. 1b).

The difference in the proportion of patients declared for either oral or LAI formulation at each stage of the process pathway, and the gap at the prescribe stage, was further explored by assessing whether physicians felt less stress, or more/no difference in stress when prescribing the two formulations. At all stages, the mean differences of proportions between patients declared for oral formulation and patients declared for LAI formulation was statistically significantly different according to physician stress levels. Notably, when physicians indicated feeling less stress, the mean proportion of patients declared was broadly similar for both formulations at each stage of the process pathway; although a small difference remained at the prescription stage (oral 48.8%, LAI 34.1%; Fig. 1c).

Regression analysis confirmed that the proportion of patients for which an antipsychotic was prescribed was significantly associated with treatment formulation (oral or LAI) and the stages of the formulation pathway, and that the interaction between formulation and stress level was significant (*p* < 0.001). If the physicians felt no or more stress, the mean difference in patient proportions for oral and LAI formulations, at all stages of the formulation pathway, was statistically significant (adjusted mean: 27.9, 95% CI: 24.9–31.0%, *p* < 0.001), whereas there was no statistical difference when physicians felt less stress (adjusted mean 3.1, 95% CI: − 5.1–11.3%, *p* = 0.453).

### Potential side effects

**Fig. 2:**
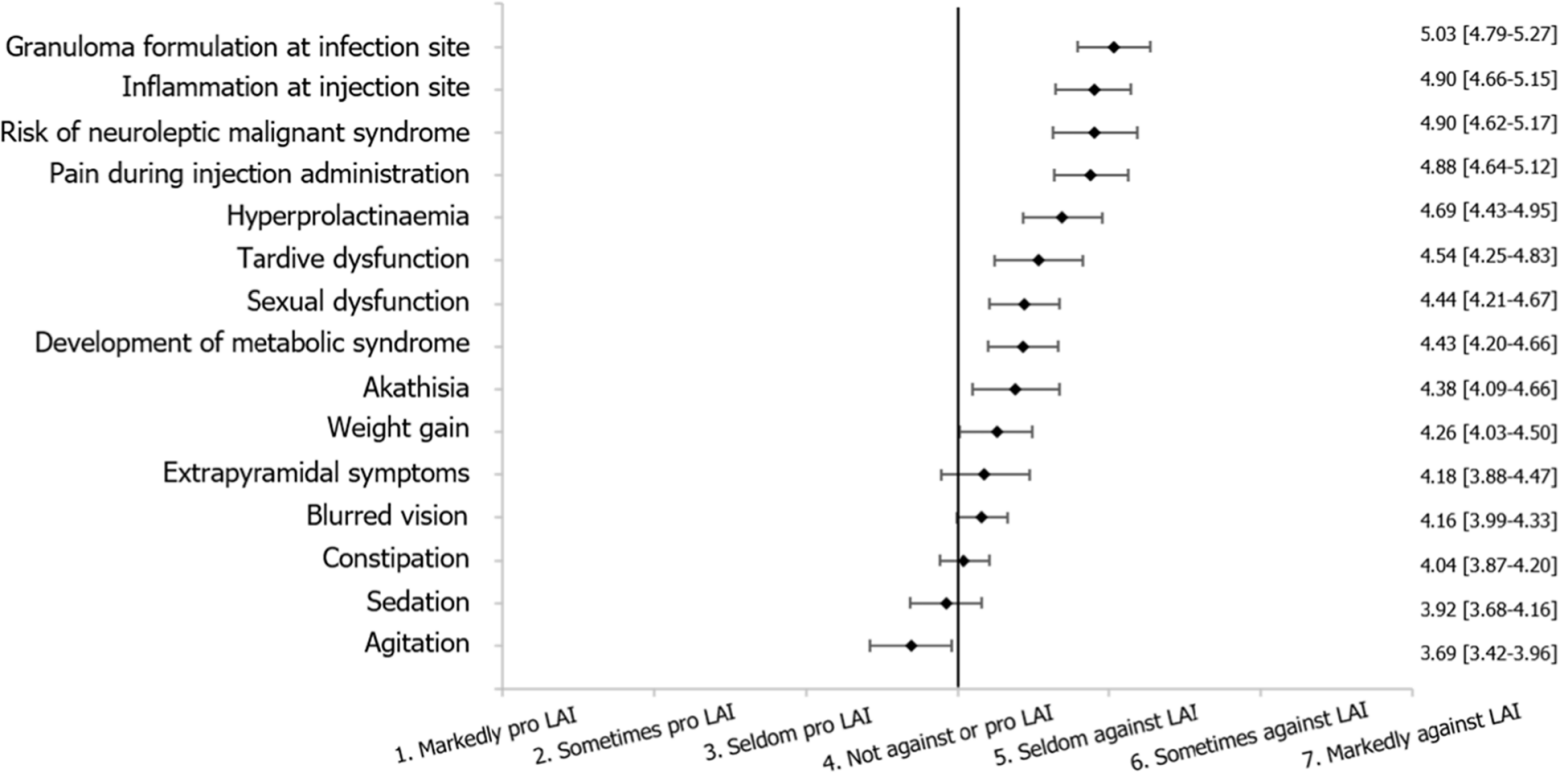
Influence of Potential Side Effects on the Initiation of Long-Acting Injectable Treatment. Mean and 95% confidence intervals are shown. Physicians scored the influence of potential side effects on initiation of LAI antipsychotic treatment with respect to a 1–7 scale (1 = “markedly pro LAI”, 2 = “sometimes pro LAI”, 3 = “seldom pro LAI”, 4 = “neither against nor pro LAI”, 5 = “seldom against LAI”, 6 = “sometimes against LAI”, 7 = “markedly against LAI”). LAI, long-acting injectable

The influence of specific side effects on the physicians’ decision to initiate LAI treatment was rated on a 1–7 scale. The four potential side effects with the highest mean scores (representing a barrier to initiating LAI treatment) included three side effects related to injection: granuloma formation at injection site (5.03 [95% CI: 4.79–5.27]), inflammation at the injection site (4.90 [4.66–5.15]), and pain during injection (4.88 [4.64–5.12], Fig. 2). Risk of neuroleptic malignant syndrome also scored comparably highly (4.90 [4.62–5.17]).

### Principal component analysis

Of the 34 original items relating to general aspects and efficacy of LAI treatment, 13 were found to have insufficient item-item correlation (Pearson correlation matrix coefficient < 0.40) and were excluded from the analysis. Sampling adequacy was confirmed (Kaiser-Meier-Olkin sampling adequacy measure 0.83) and the Bartlett’s test of sphericity was statistically significant (*p* < 0.001), indicating appropriateness of factor analysis. For the remaining 21 items, the PCA identified six separable factors with good internal consistency, which together explained 66.1% of the variance in the physician feedback (Table 2). Factor 1, “barriers” (31.4% of the variance), comprised eight items indicating prescribers disagree with the use of LAI relating to several practical and other barriers to LAI treatment. Factor 2, “injection specific barriers” (11.5% of variance), presented additional barriers to LAI prescription more specifically linked to the injection formulation per se. Factor 3, “balancing evidence of benefits” (7.5% of variance), included items on the comparative efficacy of LAIs and oral antipsychotics, patient autonomy and symptom reduction. Factor 4, “positive expectations” (6.4% of variance), described three items in agreement with LAI treatment initiation. Factor 5, “influence of colleagues” (4.8% of variance), and Factor 6, “needle fear/anxiety” (4.5% of variance), comprised two items each.

**Table 2:** Factors Identified by PCA Relating to Physicians’ Scoring in Agreement/Disagreement with LAI Formulation Use

Questionnaire Item	Disagree (against LAI use, %)	Neither (%)	Agree (pro-LAI use, %)
**Factor 1 – “Barriers” α = 0.87 31.4% of variance**			
Reduced degree of prescriber autonomy (e.g. restricted formulary or special application necessary to start LAI)	35.3	51.5	13.2
Higher loading dose or a booster injection required first	28.7	61.8	9.5
Problems with LAI cost and/ or reimbursement	43.4	45.6	11.0
Low test dose required first to check for tolerability	29.4	50.0	20.6
Oral supplementary treatment required over the first 2–3 weeks	37.5	47.1	15.4
Limitation to a particular injection frequency (i.e. only possible every 4 weeks and not flexible frequency)	39.7	41.2	19.1
Risk of increasing stigma faced by the patient	29.4	52.2	18.4
Therapeutic effect of the first injection is delayed	44.1	43.4	12.5
**Factor 2 – “Injection-specific barriers” α = 0.72 11.5% of variance**			
Lack of availability of a nurse	41.2	51.5	7.4
Choice of only gluteal for injection site	47.8	41.2	11.0
Necessity of reconstitution of the compound before it is injected	39.0	52.9	8.1
**Factor 3 – “Balancing evidence of benefits” α = 0.60 7.5% of variance**			
Evidence from clinical trials for efficacy of LAIs compared with oral antipsychotics	5.9	22.1	72.1
Preserving the patient’s autonomy	25.0	32.4	42.6
EFFICACY: Symptom reduction	5.2	31.6	63.2
**Factor 4 – “Positive expectations” α = 0.63 6.4% of variance**			
Current gastrointestinal disease with absorption problems^a^	9.6	23.7	66.7
Patient request for LAI^a^	0.7	9.6	89.6
EFFICACY: Relapse reduction	2.9	7.4	89.7
**Factor 5 – “Influence of colleagues” α = 0.91 4.8% of variance**			
Negative attitude of peer psychiatrist colleagues in your department	14.7	69.9	15.4
Negative attitude of other colleagues in your clinical team	13.2	71.3	15.4
**Factor 6 – “Needle fear/anxiety” α = 0.77 4.5% of variance**			
Anticipatory anxiety for pain	54.4	37.5	8.1
Patient’s fear of needles	69.9	22.8	7.3

^a^Denotes 1 missing answer. α denotes Cronbach’s alpha as a measure of internal consistency. Physicians were asked: “To what extent do the following general aspects affect your decision to initiate (or not initiate) LAI treatment?” Items within factors are the aspects considered and their amount of agreement/disagreement with that aspect. LAI, long-acting injectable

### Regression analysis

An unadjusted (univariate) linear regression analysis was performed on the proportion of patients diagnosed with schizophrenia evaluated by physicians as willing to accept LAI antipsychotics treatment (32.3 ± 18.4%). All six Factors identified from the PCA and certain physician characteristics were considered (Table 3). Physician age, country, and years of experience, as well as Factor 2 (injection-specific barriers) and Factor 5 (influence of colleagues) were significantly associated with the proportion of patients declared by physicians as willing to accept LAI antipsychotics treatment (*p* < 0.05, from univariate regression analyses). These variables were entered into a final adjusted (multivariate) linear regression model. From this model, the age, country, and physicians’ experience were no longer associated with patient’s willingness to accept LAI treatment (Table 3). The proportion of patients that physicians declared as willing to accept LAI treatment were significantly positively associated with the positive attitude of the physician’s colleagues (co-efficient 3.67; *p* = 0.016).

**Table 3:** Linear Regression Analysis of the Proportion of Patients Willing to Accept LAI Treatment

	Univariate			Multivariate		
	Regression coefficient	Standard Error	***p***-value	Coefficient	Standard Error	***p***-value
**Constant**	–	–	–	23.25	11.44	0.044
**Factor 1** **“Barriers”**	1.35	1.58	0.395	–	–	–
**Factor 2** **“Injection-specific barriers”**	3.20	1.57	0.044	2.81	1.63	0.087
**Factor 3** **“Balancing evidence of benefits”**	1.84	1.54	0.233	–	–	–
**Factor 4** **“Positive expectations”**	1.02	1.55	0.511	–	–	–
**Factor 5** **“Influence of colleagues”**	3.59	1.53	0.020*	3.67	1.51	0.016*
**Factor 6** **“Needle fear/anxiety”**	−0.22	1.55	0.887	–	–	–
**Male**	5.42	3.44	0.117	–	–	–
**Age (years)**	0.43	0.15	0.007*	−0.12	0.37	0.745
**General adult**	−6.38	5.77	0.271	–	–	–
**University and other clinic**	2.23	3.17	0.482	–	–	–
**Years of experience**	0.43	0.16	0.011*	0.55	0.39	0.165
**Country**	2.18	0.95	0.022*	1.40	0.97	0.151

*significantly (*p* < 0.05) associated with physicians’ declared proportion of patients willing to accept LAI treatment

## Discussion

LAI antipsychotics were developed to reduce covert non-adherence with oral treatment in patients with schizophrenia and yet their rates of prescription remain low, and there is considerable variation between service providers [24]. This study aimed to evaluate current attitudes and beliefs of European physicians towards the treatment of patients diagnosed with schizophrenia with LAI antipsychotics at a time when SGA-LAIs are more commonly used in routine clinical practice. Indeed, physicians participating in the ALTO study reported that, in their opinion, FGA-LAI prescription numbers had fallen, but that their own rates of LAI antipsychotics prescription had increased in over the past 5 years; this is likely explained by the introduction of a growing number of SGA-LAIs. Our study adds to previous work that has investigated patient attitudes towards LAI and oral antipsychotics, which identified that preferences for antipsychotic formulations do not necessarily predict attitudes [33].

Our findings, that physicians were more likely to discuss oral than LAI treatment options with patients, contrast with a previous study showing that, although most antipsychotics treatment decisions were made without patient input, when patient input occurred involvement was greater for discussions about LAIs [34]. Stress was identified as a factor influencing treatment prescribing habits, as physicians who reported no increase in stress or more stress in prescribing LAIs tended to prescribe oral over LAI antipsychotics, while those who felt less stress were more likely to prescribe LAI formulations. The specific reasons why physicians felt greater or less stress when choosing LAI treatment compared to oral treatment were not explored with the questionnaire in this study, although previous studies indicate that anxieties with regards to LAIs are sometimes based on a lack of knowledge [26, 35, 36]. Additionally, physicians can also be concerned about side effects or damaging the therapeutic relationship [34].

Our results indicate that negative colleague attitudes adversely influence physician decisions to initiate LAI treatment. Additional negative influence on physicians’ decisions to initiate LAI antipsychotic formulations regarded barriers to treatment, especially around treatment associated costs and lack of control over the specifics of LAI treatment (e.g. inflexible injection frequency, injection site, delay of therapeutic effect). In line with the results presented here, Samalin et al., conducted a study in 2013 which identified negative and positive factors influencing psychiatrists’ prescription of LAI antipsychotics [37].

Ascertaining an accurate measure of patient non-adherence is challenging and both patient and physician ratings of compliance are often inaccurate [38–40]. As non-adherence rates are underestimated [41], because of the potentially devastating effects that non-adherence can have, and due to the impact on health services in terms of clinical and economic burden, schizophrenia treatment options that tackle these issues are important. LAI antipsychotic formulations offer potential advantages over their oral counterparts in terms of decreasing rates of non-adherence but they are still not universally well-received. The choice of LAI initiation, over oral formulation antipsychotics, should be a result of shared and well-informed physician and patient decision making, with discussions regarding adherence, individual patient perspectives, and sharing of accurate information [6, 42, 43].

Findings from this study may have important implications for current clinical practice and could potentially lead to more evidence-based use of LAI antipsychotics. Recently published data from the PRELAPSE trial in patients with early schizophrenia suggests that more frequent discussion on LAI antipsychotics use with prescribers across different healthcare settings could help to remove potential logistical barriers and increase the use of LAI antipsychotics [44]. This data is supported by ethnographic evidence that limited physician knowledge on LAI antipsychotics, including concerns about the pharmacological properties of LAI prescriptions, may be a barrier to their use [43].

One limitation of the ALTO study relates to potential sampling bias. The physicians’ whose attitudes were analysed as part of this study were drawn from a pool of physicians who already chose to prescribe LAI antipsychotics. As such, these physicians may have more positive attitudes towards LAI antipsychotics than the general European physician population, and therefore may be more predisposed to prescribing LAI antipsychotics. However, it is important to note that variation was still seen in the attitudes of the pool of physicians investigated here. The influence of positive, or other, colleague attitudes towards the use of LAI treatment and prescription behaviour was not assessed (only questions on the negative attitudes of colleagues were included in the questionnaire) and so this may provide an avenue for future research. Other limitations of the study include the unbalanced distribution of participants by country, as well as the inability to examine whether prescription of LAI antipsychotics was greater among physicians treating a higher number of patients with schizophrenia, or if prescription rates differed for patients of physicians in clinical practice (and the potential impact of negative influence on such decisions). Furthermore, we cannot discount the possibility that some physicians may provide a theoretical view on their prescribing attitudes, and this may not reflect their actions in everyday practice.

## Conclusions

To the best of our knowledge, the ALTO study is the first to evaluate the attitudes of a wide demographic of psychiatrists (five different countries across multiple clinical settings) and offers important insight into physician attitudes regarding the acceptance and usage of LAI antipsychotics to treat patients with schizophrenia. Our results suggest that physician attitudes can influence treatment formulation choices; therefore, it is important to understand how and why different therapeutics are perceived favourably or unfavourably in clinical practice.

## Data Availability

The datasets generated during and/or analysed during the current study are available from the corresponding author on reasonable request. The study protocol is available from AGN@lundbeck.com.

## Abbreviations

ALTO: Antipsychotic Long acTing injection in schizOphrenia
CI: Confidence interval
FGA: First-generation antipsychotic
LAI: Long-acting injectable
NIS: Non-interventional study
PCA: Principal component analysis
RCT: Randomised controlled trial
SGA: Second-generation antipsychotic
